# Parkin characteristics and blood biomarkers of Parkinson’s disease in WPBLC study

**DOI:** 10.3389/fnagi.2025.1511272

**Published:** 2025-02-26

**Authors:** Haijun He, Xi Xiong, Yi Zheng, Jialong Hou, Tao Jiang, Weiwei Quan, Jiani Huang, Jiaxue Xu, Keke Chen, Jingjing Qian, Jinlai Cai, Yao Lu, Mengjia Lian, Chenglong Xie, Ji Luo

**Affiliations:** ^1^Department of Neurology, The Affiliated Huzhou Hospital, Zhejiang University School of Medicine (Huzhou Central Hospital), Huzhou, China; ^2^Department of Neurology, The First Affiliated Hospital of Wenzhou Medical University, Wenzhou, China; ^3^Department of Neurology, The First People’s Hospital of Wenling, Taizhou, China; ^4^Department of Neurology, Yuhuan City People’s Hospital, Taizhou, China; ^5^Department of Physiology, School of Medicine, National and Kapodistrian University of Athens, Athens, Greece

**Keywords:** Parkin, blood biomarkers, mitophagy, Parkinson’s diseaseas, bioinformatics

## Abstract

**Background:**

The exact mechanisms of PD are unclear, but Parkin-mediated mitophagy dysfunction is believed to play a key role. We investigated whether blood levels of Parkin and other biomarkers are linked to the risk of developing PD.

**Methods:**

Baseline blood measures of Parkin and other biomarkers, including Homocysteine, carcinoembryonic antigen, Urea, total proteins, total cholesterol, creatine kinase, and albumin, were collected from 197 clinically diagnosed Parkinson’s disease participants and 107 age-matched healthy controls in Wenzhou Parkinson’s Biomarkers and Living Characteristics study. We conducted bioinformatics analysis using three datasets from the GEO database: GSE90514 (Cohort 1: PD = 4, HC = 4), GSE7621 (Cohort 2: PD = 16, HC = 9), and GSE205450 (Cohort 3: PD = 69, HC = 81).

**Results:**

Using a bioinformatic approach, we identified dysregulated biological processes in PD patients with PRKN mutations. Compared to controls, significant abnormalities were observed in blood levels of Parkin, Hcy, total proteins, urea, albumin, and CEA in PD patients. A model incorporating Parkin, Hcy, total proteins, and urea effectively distinguished PD from healthy controls, achieving a higher accuracy (AUC 0.841) than other biomarker combinations. Gene set enrichment analysis suggested that pathways such as PINK1-Parkin-mediated mitophagy, urea cycle, cysteine degradation, and riboflavin metabolism may be involved in PRKN mutation. Additionally, the link between Parkin and PD was partially mediated by CEA and albumin, not by Hcy, total proteins, or urea.

**Conclusion:**

Our findings indicate that blood Parkin levels may be a minimally invasive biomarker for PD diagnosis. The model, which included Parkin, Hcy, total proteins, and urea, effectively distinguished PD from HC with greater accuracy.

## Introduction

Two centuries have elapsed since James Parkinson announced his seminal work “An Essay on the Shaking Palsy” in 1817, describing the clinical characteristics of this disease that later came to endow his name (Parkinson, 2002; Bloem et al., 2021). But to now, there is still no precise and widely used laboratory testing for Parkinson’s disease (PD) diagnosis. Currently, the diagnosis of PD mainly relies on symptom-driven performance, which delays the detection of the earliest phases of the disease. Moreover, even when such criteria are rigorously executed, the proportion of misdiagnosis is still high resulting from substantial clinical overlap among Parkinsonian syndromes (Armstrong and Okun, 2020). Thus, reliable diagnostic biomarkers are urgently needed to efficiently manage PD. Evidence indicates the potential diagnostic and prognostic merit of cerebrospinal fluid (CSF) and blood biomarkers authentically mirroring the pathogenesis of PD, such as *α*-synuclein isoforms, lysosomal enzymes, amyloid and tau pathology markers, and neurofilament light chain (NFL) (Parnetti et al., 2019; Bouthour et al., 2019; Ashton et al., 2020). Compared to the CSF fluid, blood-based biomarkers are also under far-ranging investigation because they would provide a minimally invasive option for early and differential diagnosis of PD versus atypical Parkinsonian disorders and disease monitoring.

Although the mechanisms of PD are unclear, mitochondrial dysfunction and quality control imbalance are thought to have key roles in this process (Malpartida et al., 2021). Notably, an early-onset form of PD is associated with mutations in the PINK1 kinase and Parkin ubiquitin ligase genes (Farrer, 2006; Valente et al., 2004). Exploring the characteristics of genes mutated in hereditary PD type sheds light on disease etiology and reveals new pathways in cell biology (Blauwendraat et al., 2020). Among them, PINK1 and Parkin, which usually work together in the same pathway, are involved in the clearance of damaged mitochondria in PD-related cultured cells and animal models (Nguyen et al., 2016; Pickrell and Youle, 2015). Moreover, the drop of dopaminergic neurons in the substantia nigra pars compacta (SNpc) and the motor defect observed in aged Parkin−/− mice indicate the Parkin-mediated biological pathway facilitates this phenotype (Sliter et al., 2018). These findings highlighted the underlying value of considering the level of Parkin when implementing blood biomarkers in the diagnostic workup of PD.

The prevailing hypothesis for PD associated with *PRKN* mutations (also known as *PARK2*) is that a decrease in Parkin activity alters the mitophagy machinery and results in increased *α*-synuclein aggregation and accumulation in the lysosomes (Wang et al., 2022). Several studies suggest that loss of function mutations in the *PRKN* gene that encodes the Parkin may promote α-synuclein-mediated Lewy body inclusion formation, further suggesting the importance of studying this target as a biomarker of PD (Madsen et al., 2021; Yasuda and Mochizuki, 2010). However, it is still not a clinically helpful biomarker for PD, measurements of Parkin in biofluids from well-clinically characterized subjects may provide additional insight into whether Parkin ubiquitin ligase may be deregulated in PD cases. Thus, it would be vital to carry out research to monitor Parkin levels and determine its utility as a biomarker of PD screening. In this study, we aimed to test whether Parkin levels were elevated in PD subjects and whether levels were associated with PD status. We hypothesized, based on the previous literature (Wang et al., 2022; Yasuda and Mochizuki, 2010; Madsen et al., 2021; Qian et al., 2024) and our results, that blood Parkin would be a superior marker for PD diagnosis.

## Methods

The cross-sectional study is rated Class III because of the case–control design and the absence of diagnostic uncertainty of PD in the included patients.

### Participants

The WPBLC cohort (Wenzhou Parkinson’s Biomarkers and Living Characteristics study, included 197 PD patients and 107 age-matched healthy controls from the First Affiliated Hospital of Wenzhou Medical University, March 2018–October 2022, details are available in Supple information 1) included two subsets: subset 1 with 55 Parkinson’s disease (PD) patients (patients diagnosed with idiopathic Parkinson’s disease) and 50 healthy control (HC) participants, who were inpatients tested with 165 additional blood biomarkers, and subset 2 with 142 PD patients and 57 HC participants, who lacked these extra biomarkers.

### Clinical neuropsychological evaluation

At the screening visit, standardized methods for the acquisition of study data included the Unified Parkinson’s Disease Rating Scale (UPDRS) (Fahn et al., 1987) and Hoehn-Yahr staging (Hoehn and Yahr, 1967) to evaluate the motor symptoms and progression stage of PD. The Chinese Mini-Mental State Examination (MMSE) was used for cognitive assessment, with cutoff scores adjusted to: ≤ 17 for illiterates, ≤ 20 for primary school graduates, and ≤ 24 for those with postsecondary education or higher (Katzman et al., 1988; Cui et al., 2011). Emotional aspects were assessed using the Hamilton Depression Rating Scale-17 (HAMD) and Hamilton Anxiety Rating Scale (HAMA), with scores ≥7 indicating possible depression or anxiety. The REM Sleep Behavior Disorder Questionnaire-Hong Kong (RBDQ-HK) identified REM sleep behavior disorder (RBD) with a cutoff of >18 points (Li et al., 2010). The Activity of Daily Living Scale (ADL) is used to collectively assess fundamental skills required to independently care for oneself, such as eating, bathing, and mobility. All the examinations were done in the “on” state of the disease.

### Blood Parkin and other biomarkers measurement

**Figure 1:**
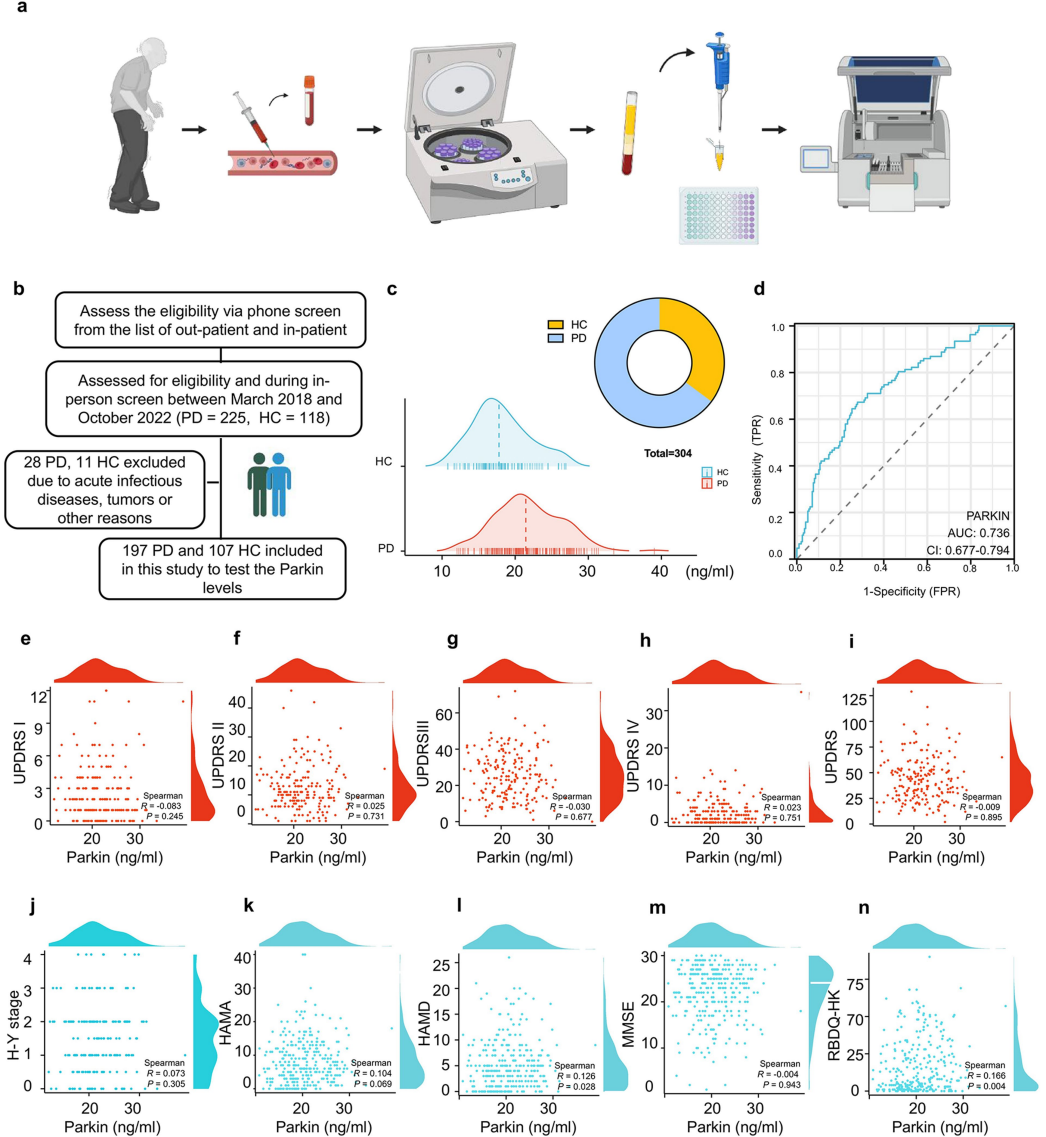
Recruitment of participants, blood sample processing, and the efficacy of Parkin protein levels in diagnosing and correlating with PD symptoms. **(A)** A flow chart outlines the blood Parkin examination process for study participants. **(B)** Eligibility assessments resulted in 197 PD and 107 HC subjects being included. **(C)** A mountain map illustrates the distribution of Parkin levels in PD and HC individuals. **(D)** ROC analysis evaluated Parkin levels’ ability to differentiate between PD and HC, with the AUC value reported. **(E–N)** Nonlinear correlation analyses were conducted between Parkin levels and neuropsychological assessment scores, with Spearman correlation coefficients calculated.

Figure 1A shows the flow chart of blood Parkin examination for every individual enrolled in the study. The detailed measurements of Parkin have been previously presented (Qian et al., 2024). Plasma samples were collected via venous blood centrifugation (3,000 × g for 10 min) at 4°C and frozen at −80°C until analysis. Blood was drawn using an EDTA anticoagulant tube and centrifuged within 1 h. A total of 304 participants’ samples (197 PD and 107 HC) were analyzed for Parkin using an ELISA (Jianglai Biotechnology Company, Shanghai, China, No#. JL11195). Additionally, 234 of these samples (148 PD and 86 HC) were analyzed for *α*-syn oligomers (asy-no) and phosphorylated α-syn (p-asyn) using ELISA (Jianglai Biotechnology Company, Shanghai, China, No. JL12589 and JL41188). A blinded laboratory technician processed the samples according to the manufacturer’s instructions. Eighty microliters of standard solution and 20 microliters of 5× diluted samples were added to 96-well plates. Then, 100 microliters of antibody-horse radish peroxidase conjugate (MyBioSource, United States) was added to each well, covered with an adhesive strip, and incubated for 60 min at 37°C. After four washes, the plates were incubated with tetramethylbenzidine substrate for 15 min at 37°C, then the reactions were stopped with H2SO4. Absorbance was measured at 450 nm, with all samples run in triplicate.

Inpatients were assessed for 165 blood biomarkers, sourced from the First Affiliated Hospital of Wenzhou Medical University. Of these, 66 biomarkers were selected for analysis, while 99 were excluded due to insufficient subjects. Details on the blood Parkin and other 66 blood biomarkers are available in Supplementary Table 1.

### Statistical analyses

Continuous variables were evaluated for normality with the Kolmogorov–Smirnov test, histogram, and Q-Q plot. Normally distributed variables were reported as mean (Standardized deviation, SD) and analyzed with a two-sample t-test, while abnormally distributed variables were reported as median [interquartile range, IQR] and analyzed using the Mann–Whitney U test. Categorical variables were presented as counts (percentages) and compared with the Chi-square test. Associations between biomarkers and neuropsychological scales were assessed using Spearman rank correlation analysis. The Random Forest (RF) classifier and Least Absolute Shrinkage And Selection Operator (LASSO) regression were employed to identify effective blood biomarkers for distinguishing PD from HC participants. The RF classifier, utilizing the “randomForest” R package with 100 trees, ranked blood biomarkers based on their importance using the Gini index. LASSO regression was performed on standardized blood biomarker levels using the “glmnet” package. The predictive power of the biomarkers, both individually and together, was evaluated through the area under the curve (AUC) from ROC curves, with differences assessed using DeLong statistics. Some combined models were tested in Cohort 1 and validated in Cohort 2 to assess their stability.

Blood biomarkers were divided into four quartiles (Q1-Q4) as categorical variables, and a trend test was conducted using the median values of each quartile. Weighted quantile sum (WQS) regression was used to assess the overall effects of these biomarkers on PD risk. The R package “gWQS” calculates the WQS index from the weighted sums of individual biomarkers. The WQS index (0 to 1) indicated the combined levels of blood biomarkers, with significant components identified by their weights. To evaluate the joint effects and dose–response relationships of individual biomarkers on PD risk, while controlling for others, Bayesian kernel machine regression (BKMR) was utilized. Mediation analyses were conducted using the R package “mediation” with the quasi-Bayesian Monte Carlo method and 1,000 simulations based on normal approximation. The direct effect (DE) indicated the impact of blood biomarkers on PD risk without mediation, while the indirect effect (IE) reflected their impact through a mediator. The proportion of mediation was calculated as IE divided by the total effect (TE). Statistical analyses were performed in R version 4.3, with significance set at *p* < 0.05.

### Bioinformatics analysis

#### GEO datasets acquisition

We retrieved three datasets from the Gene Expression Omnibus (GEO) database: GSE90514 (Cohort 1: PD = 4, HC = 4), GSE7621 (Cohort 2: PD = 16, HC = 9), and GSE205450 (Cohort 3: PD = 69, HC = 81). GSE90514 used the GPL11154 Illumina HiSeq 2000 platform, GSE7621 used the GPL570 Affymetrix Human Genome U133 Plus 2.0 Array, and GSE205450 used the GPL24676 Illumina NovaSeq 6,000 platform. We downloaded the expression matrices and annotation data, then normalized and log2-transformed the matrices using R version 4.3.

#### Identification and visualization of DEGs

We merged Cohort 2 and Cohort 3 using 15,596 shared genes and performed batch correction using the “SVA” package to obtain Cohort C. We conducted the differential analysis using the “LIMMA” package on Cohort 1 and Cohort C and filtered for DEGs with *p* < 0.05 and |log FC| (fold change) > 1.5 (Law et al., 2014). We obtained 322 DEGs in Cohort 1 and 16 DEGs in Cohort C, which were visualized using volcano plots and heatmaps.

#### Enrichment analyses

For gene enrichment analyses, we used the “ClusterProfiler” package to filter for relevant pathways with a threshold of *p* < 0.05 and False Discovery Rates (FDR) < 0.1 (Yu et al., 2012). In Cohort 1, 82 significant gene ontology (GO) pathways were enriched (Gene Ontology Consortium, 2015), and we have selected 10 pathways for presentation.

#### Gene set enrichment analysis (GSEA)

Gene Set Enrichment Analysis was conducted using the GSEA software (version 3.0) obtained from the GSEA website.^1^ GSEA was used to analyze the enrichment of all detected genes in Cohort 1 for KEGG and Reactome Pathways. Finally, we found 34 enriched KEGG pathways and 99 enriched Reactome Pathways (Wixon and Kell, 2000).

#### Gene set variation analysis (GSVA) of GO enrichment

GSVA was performed using the “GSVA” package to calculate the enrichment scores of each sample in Cohort 1 for KEGG pathways (Hänzelmann et al., 2013). We subsequently used the “LIMMA” package to identify 15 differential pathways (8 upregulated and 7 downregulated) and generated volcano plots to visualize the differentially regulated pathways.

#### Analysis of key genes in the train cohort and the test cohort

We divided Cohort C into a Training set and a Test set. In the Training set, we identified 16 genes with statistical significance (*p* < 0.05, |log FC| > 1.5). We used Lasso regression to select 6 key genes: *NUP210L*, *SLCO4A1*, *AMBN*, *GPD1*, *NTRK1*, and *HBB,* for modeling. We then validated the model in the test set and calculated the Area Under The Curve (AUC) value.

## Results

This paper addressed three hypotheses: First, we evaluated blood Parkin levels in PD participants to assess its potential as a diagnostic biomarker. Second, we investigated whether other blood biomarkers may be viable tools for distinguishing PD patients with PRKN mutations based on bioinformatic analysis. Third, we explored the relationship between Parkin levels and other blood biomarker profiles in PD by quantifying their associations in our cohort.

### Baseline demographics, disease characteristics of the cohorts

Table 1 shows the demographic data for the WPBLC cohort investigated here with 197 PD and 107 HC subjects. For patients with PD and HC information, similar to previous reports of this cohort at baseline, mean age at onset, sex ratio, height, weight, BMI status, education level, disease duration, smoker proportion, drinker ratio, and frequency of diabetes mellitus were matched. By contrast, PD patients were characterized by much more serious UPDRS, HAMD, HAMA, RBDQ-HK, and ADL scores compared to the controls. Accordingly, the clinical phenotypes of advanced PD symptoms, i.e., falls, dyskinesia, on–off phenomenon, and cognitive impairment were also displayed in Table 1.

**Table 1:** Basic characteristics of PD patients and healthy controls.

Characteristics	HC (*N* = 107)	PD (*N* = 197)	*p* value
Age (years)	65.0 [59.0;69.0]	67.0 [61.0;72.0]	0.146
Sex			0.066
Female	62 (57.9%)	91 (46.2%)	
Male	45 (42.1%)	106 (53.8%)	
Height (cm)	161 (7.05)	161 (8.33)	0.579
Weight (kg)	63.5 (9.23)	61.7 (10.5)	0.124
BMI (kg/m2)	24.4 (2.86)	23.8 (3.22)	0.131
BMI Group			0.567
<24	51 (47.7%)	105 (53.3%)	
24–28	12 (11.2%)	23 (11.7%)	
>28	44 (41.1%)	69 (35.0%)	
Education (years)	5.00 [0.00;6.50]	4.00 [0.00;7.00]	0.837
Disease History (years)	-	3.00 [2.00;7.00]	-
Smoker			0.308
Current	16 (15.0%)	25 (12.7%)	
Former	2 (1.87%)	11 (5.58%)	
Never	89 (83.2%)	161 (81.7%)	
Drinker			0.846
Current	17 (15.9%)	35 (17.8%)	
Former	4 (3.74%)	6 (3.05%)	
Never	86 (80.4%)	156 (79.2%)	
HP			0.001
No	52 (48.6%)	135 (68.5%)	
Yes	55 (51.4%)	62 (31.5%)	
DM			0.807
No	91 (85.0%)	164 (83.2%)	
Yes	16 (15.0%)	33 (16.8%)	
LEDD	-	375 [300;581]	-
Related scales			
UPDRS	-	39.0 [28.0;53.0]	-
I	-	2.00 [1.00;4.00]	-
II	-	11.0 [8.00;16.0]	-
III	-	24.0 [15.0;34.0]	-
IV	-	2.00 [0.00;4.00]	-
H-Y stage	-	2.50 [1.50;3.00]	-
MMSE	24.0 [20.5;26.0]	23.0 [18.0;27.0]	0.164
HAMD	3.00 [0.00;5.00]	5.00 [3.00;9.00]	<0.001
HAMA	4.00 [1.00;7.00]	9.00 [5.00;13.0]	<0.001
RBDQ-HK	4.00 [1.00;9.50]	15.0 [3.00;34.0]	<0.001
ADL	20.0 [20.0;20.0]	26.0 [21.0;34.0]	<0.001
Complications			
Fall			-
No	-	154 (84.6%)	
Yes	-	28 (15.4%)	
Dyskinesia			-
No	-	170 (92.9%)	
Yes	-	13 (7.10%)	
On–off			-
No	-	137 (74.9%)	
Yes	-	46 (25.1%)	
Cognitive impaired			0.161
No	72 (67.3%)	115 (58.4%)	
Yes	35 (32.7%)	82 (41.6%)	

Continuous variables were assessed for normality using Kolmogorov–Smirnov test, to variables on normal distribution, results were expressed as the mean ± standard deviation (SD) and compared using the student’s t-test; while data on non-normal distribution, variables were exhibited as median [IQR] and compared using the Mann–Whitney U-test. Categorical variables were listed as number (percentage) and compared using the chi-squared test.

HC: Healthy Control; PD: Parkinson’s disease; BMI: Body Mass Index; Smoker (Current: people who have smoked continuously or cumulatively for 6 months or more and still smoke at the time of the survey; Former: people who have smoked for more than 6 months and did not smoke at the time of the survey; Never: people who have smoked for less than 6 months throughout their lives); Drinker (Current: people who have drunk alcohol continuously or cumulatively for 6 months or more and still drink at the time of the survey; Former: people who have drunk alcohol for more than 6 months and did not drink at the time of the survey; Never: people who have drunk alcohol for less than 6 months throughout their lives); HP: hyper blood pressure; DM: diabetes mellitus; LEDD: Levodopa Equivalents. UPDRS: unified Parkinson’s disease rating scale; MMSE: Mini-Mental State Examination; HAMD: Hamilton Depression Scale; HAMA: Hamilton Anxiety Scale; RBDQ-HK: REM sleep behavior Disorder questionnaire-Hong Kong; ADL: Activity of Daily Living Scale; Fall: subjects had a fall within a year; Dyskinesia: subjects with abnormally increased involuntary movements; On–off: subjects with drug effect fluctuation after long-term use of levodopa. Cognitive impaired: subjects with MMSE scores lower than cutoff value.

### Parkin is elevated in the blood of PD patients

Three hundred and four participants met the inclusion criteria for the initial group analysis (Figure 1B): 100% provided samples for Parkin measurement from blood. Comparing blood Parkin levels across diagnostic groups, the median concentration was 21.458 ng/mL in PD subjects, and 17.789 ng/mL in HC (Figure 1C). Then, assessing the utility of Parkin levels to discriminate between clinically defined idiopathic PD and HC, we found an area under the ROC curve (AUC) of 0.736 (95% CI: 0.677 to 0.794) for Parkin (Figure 1D), indicating Parkin is a moderately suitable diagnosis marker for PD. To test whether Parkin levels are correlated with clinical motor features. In 197 participants who had blood Parkin and motor evaluation drawn simultaneously, Parkin was not correlated with UPDRS part I (*r* = −0.083, *p* = 0.245), UPDRS part II (*r* = 0.025, *p* = 0.731), UPDRS part III (*r* = −0.030, *p* = 0.677), UPDRS part IV (*r* = 0.023, *p* = 0.751), total UPDRS (*r* = −0.009, *p* = 0.895), and H-Y stage (*r* = 0.073, *p* = 0.305) based on the Spearman correlation analysis (Figures 1E–J). Next, we examined associations between Parkin and neuropsychological scales. We found that Parkin concentrations correlated with baseline HAMD and RBDQ-HK, while not with baseline HAMA and MMSE (Figures 1K–N). Notably, the UPDRS part III score may be influenced by age, sex, disease duration, and LEDD. Hence, we evaluated associations between Parkin measures and motor performance in models adjusting for these variables and found the relationship remained not significant.

Moreover, in patients with Parkinson’s disease, pathological accumulation of *α*-synuclein in the brain occurs prior to the onset of motor symptoms. Increased α-syn, such as asy-no and p-asyn in the blood has been proposed as biomarkers of PD diagnosis (Atik et al., 2016). Spearman’s correlation analysis was performed to determine whether Parkin correlated to the asy-no and p-asyn concentrations. We found that Parkin was significantly positively correlated to asy-no (*r* = 0.453, *p* < 0.001, Supplementary Figure 1A) and p-asyn (*r* = 0.428, *p* < 0.001, Supplementary Figure 1B), indicating the probable interaction of Parkin and a-syn in the PD pathogenesis. The ROC analysis showed that a blood Parkin cutoff value of 19.141 ng/mL had a sensitivity of 78.6% and a specificity of 83.7% for distinguishing between PD and HC (Figure 1D). Next, all subjects were divided into Parkin positive (+) and negative (−) according to this cutoff value (Supplementary Figure 1C). Baseline levels of asy-no (Supplementary Figure 1D) and p-asyn (Supplementary Figure 1E) were higher in patients with Parkin-positive (+) subjects compared to the negative (−) groups. Whereas the scores of UPDRS part I-IV and total were similar between Parkin (+) and (−) groups (Supplementary Figures 1F–J), we did not find any association of Parkin status with motor scales. Then, to test the association of Parkin status with neuropsychological scales in PD subjects, we found higher HAMA, HAMD, RBDQ-HK, and ADL performance in the PD Parkin (+) groups (Supplementary Figures 1K–O).

### Data mining of PD patients with PRKN mutations by bioinformatic analysis

**Figure 2:**
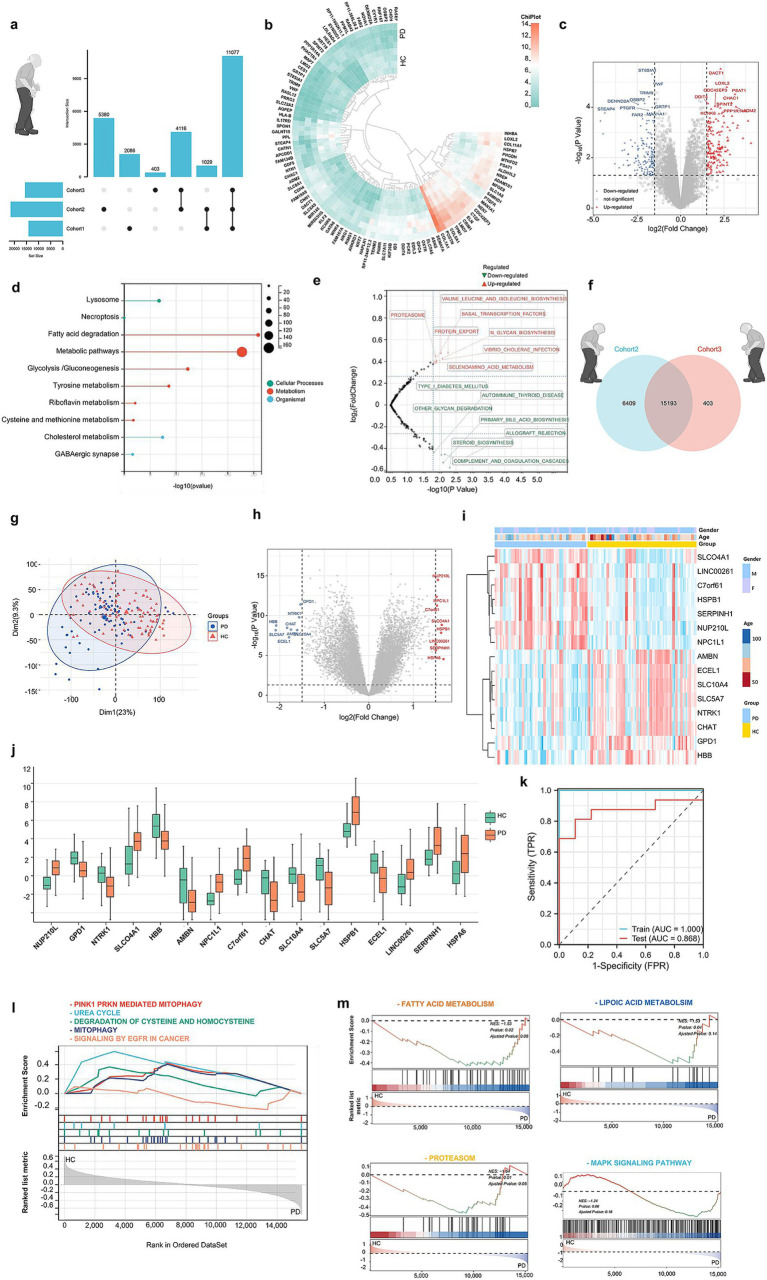
Data mining of PD patients with PRKN mutations by bioinformatic analysis. **(A)** UPSet plot showing unique and shared genes across Cohorts 1, 2, and 3. **(B)** Heatmap of the top 464 differentially expressed genes (DEGs) in PD vs. HC for Cohort 1, selected with *p* < 0.05 and |logFC| > 1.5, including 4 PD and 4 HC samples. **(C)** Volcano plot of the top 464 DEGs in PD vs. HC for Cohort 1. **(D)** GO analyses revealed common perturbed pathways in PD subjects with PRKN mutations. **(E)** GSVA of Cohort 1 shows upregulation in 8 metabolic pathways, such as proteolysis and protein export, and downregulation in 7 pathways. **(F)** A Venn plot reveals 15,193 genes common to Cohort 2 and 3. **(G)** Cohorts 2 and 3 were combined and batch-corrected with the “SVA” package, resulting in Cohort C, comprising 85 PD and 90 HC samples. **(H)** Heatmap of average expression levels for 16 significant DEGs in Cohort C’s training set. **(I)** Volcano plot illustrating DEGs between PD and HC in the same training set, highlighting 16 significant DEGs. **(J,K)** Bar graph of the 16 DEGs’ expression levels in the training set, confirming observed expression patterns. Lasso regression identified 6 genes (NUP210L, SLCO4A1, AMBN, GPD1, NTRK1, HBB) from 16 DEGs, resulting in a model with an AUC of 0.868 in the test set. **(L,M)** GSEA in Cohort C showed enrichment in 186 KEGG pathways and 1,586 Reactome pathways, highlighting the top 5 Reactome and 4 KEGG pathways.

We analyzed expression datasets from patients with PD with *PRKN* mutation (GSE90514, GSE7621, and GSE205450) archived in GEO datasets to define the omics features associated with the disease. A total of three cohorts comparing PD patients with *PRKN* mutations to healthy controls were found, which referred to changes in the transcriptional levels from the skin fibroblasts, substantia nigra, caudate, and putamen biospecimen, respectively. We identified thousands of differentially expressed genes (DEGs) that were implicated in these three Cohorts (Figure 2A). For cohort 1 (González-Casacuberta et al., 2018), a similar analysis of DEGs using high-quality bulk RNA sequencing (RNA-seq) data from the GSE90514. Heat map showing expression of DEGs in every sample (Figure 2B). Moreover, the volcano plot depicts the top upregulated and downregulated genes in PD subjects with *PRKN* mutations compared to controls (Figure 2C). Metabolism and Protein GO analyses revealed common perturbed pathways in PD subjects with *PRKN* mutations, including lysosome, Fatty acid degradation, Glycolysis, Tyrosine metabolism, and Cholesterol metabolism et al. (Figure 2D). Next, Gene Set Variation Analysis (GSVA) as a non-parametric, unsupervised method for estimating the variation of pathway activity through the samples of an expression data set (Hänzelmann et al., 2013). In PD subjects with *PRKN* mutations, GSVA showed that a gradual increase in the Proteasome, Protein Export, Selenoamino acid metabolism, N-glycan biosynthesis pathways, *et al* (Figure 2E). These results suggested that the wide bioenergy metabolism turbulences were observed in PD subjects with *PRKN* mutations.

Next, the Venn diagram illustrates the overlap of genes between Cohort 2 and Cohort 3 (Figure 2F). As shown in Figure 2G, there was a mild separation of PD subjects from healthy controls on the PCA score plot, indicating the reasonable to pool Cohort 2 and Cohort 3. Notably, the volcano plot and heatmap displayed the primary 16 upregulated and downregulated genes in PD patients when we pooled Cohort 2 and 3 (Figures 2H,I). Importantly, based on the RNA-seq analysis, the transcriptional levels of these 16 genes were changed in the PD groups, such as *nup21ol*, *slca4a1*, *npc1l1*, *c7orf61*, *hspb1*, *serpinh1*, and *hspa6* et al. (Figure 2J). Then, we identified the promising 6 powerful genes (*NUP210L*, *SLCO4A1*, *AMBN*, *GPD1*, *NTRK1*, *HBB*) after the Lasso regression analysis. To test the selected 6 genes’ capacity to discriminate between PD and controls, the AUC was 0.868 (Figure 2K). Finally, we aimed to assess the expression of *a priori* identified gene sets and biological pathways associated with PD using Gene Set Enrichment Analysis (GSEA) in pooled cohorts 2 and 3. We adopted the Reactome analysis to describe human biological processes in PD background, and by mapping disease-associated pathways (Figures 2L,M). Notably, PINK1-PRKN MEDIATED MITOPHAGY, UREA CYCLE, DEGRADATION OF CYSTEINE AND HOMOCYSTEINE, MITOPHAGY and SIGNALING BY EGFR IN CANCER were enriched in PD, suggesting mitochondrial dysfunction and metabolic abnormalities (Figure 2L). Additionally, FATTY ACID METABOLISM, LIPOIC ACID METABOLSIM, PROTEASOM, and MAPK SIGNALING PATHWAY showed dysregulation, highlighting metabolic imbalances in PD (Figure 2M). These findings provide new insights into the molecular mechanisms underlying PD and may aid in the identification of potential biomarkers and the development of targeted therapeutic strategies.

### Discriminative accuracy of blood biomarkers for PD patients

**Figure 3:**
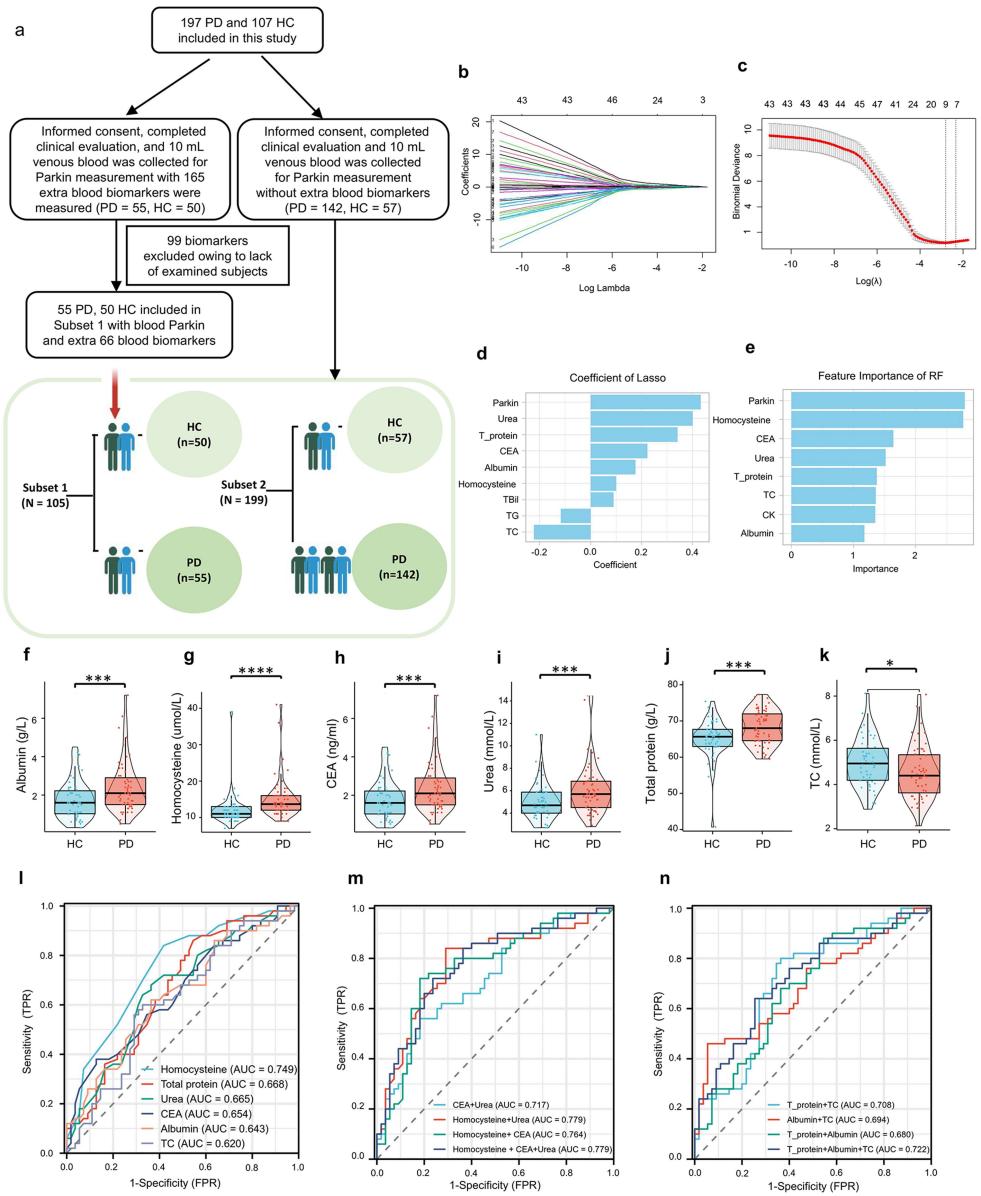
Discriminative Accuracy of blood parkin and additional blood biomarkers for PD patients. **(A)** Subjects were split into two cohorts based on extra blood tests: Subset 1 included 55 PD and 50 HC subjects with additional biomarkers, while Subset 2 comprised the remaining 142 PD and 57 HC subjects. **(B)** Blood parkin levels and biomarkers were standardized, and z-scores were used for LASSO regression analysis, with diagnosis as the dependent variable, resulting in coefficient profiles for 67 variables. **(C)** The optimal *λ* value for LASSO regression was determined using 10-fold cross-validation, with dotted vertical lines indicating values from the minimum criteria (left) and the “one standard error” criteria (right). **(D)** Nine biomarkers were selected based on the minimum λ criteria, and their LASSO coefficients are shown in the bar graph. **(E)** Feature importance for HC/PD classification was assessed using a Random Forest model with 100 decision trees, ranking the top 8 blood biomarkers displayed in a bar graph. **(F–K)** Six biomarkers were common between LASSO and Random Forest selections, while boxplots illustrated levels of six additional biomarkers in PD and HC subjects. **(L)** ROC analysis results for the six biomarkers were presented individually. **(M)** ROC analysis results for models combining blood CEA, HCY, and urea levels were presented. **(N)** ROC analysis results for models with nutrition-related biomarkers, including total protein, TC, and albumin levels, were also shown. *p < 0.05, **p < 0.01, ***p < 0.001, ****p < 0.0001.

Extract from the WPBLC cohort, 105 blood samples (subset 1: PD: 55, HC: 50) test the 66 common biomarkers (Figure 3A and Supplementary Table 1). Details for the basic characteristics of these sunsets are provided in Supplementary Table 2. Least absolute shrinkage and selection operator (LASSO) regression is an adaptation of the popular and widely used linear regression algorithm as a new mathematical prediction model to select variables in disease diagnosis (Li et al., 2022). In our study, since we have many blood biomarkers and relatively few cases, the LASSO regression analysis was applied to pick out the biomarkers most associated with PD, and the top 9 powerful variables were identified (Figures 3B–D). Moreover, we used the random forest (RF) analysis, a machine learning approach that aids in identifying several model components and quantifiable pre-simulation (Zhao et al., 2018). We trained a RF, tested its predictive accuracy and established the following 8 most promising factors in the PD diagnosis set (Figure 3E). The overlapping parts of LASSO and RF selected biomarkers were chosen for further analyses. These included Parkin, Homocysteine (Hcy), carcinoembryonic antigen (CEA), Urea, Total proteins, total cholesterol (TC), and Albumin.

As seen in Figures 3F–K and Supplementary Table 3 in supporting information, higher levels of Hcy, CEA, Urea, total proteins, and albumin were observed in PD participants; only TC displayed the opposite direction. Moreover, we used ROC analyses to assess the utility of these selected blood biomarker levels to discriminate between PD and controls (Figure 3L and Supplementary Table 4). The AUCs were 0.749 for Hcy (sensitivity = 0.84, specificity = 0.582), 0.668 for Total proteins (sensitivity = 0.86, specificity = 0.455), and 0.665 for Urea (sensitivity = 0.68, specificity = 0.636). By contrast, the discrepancy between the control and PD groups was low in the CEA (AUC: 0.654, sensitivity = 0.38, specificity = 0.873), albumin (AUC: 0.643, sensitivity = 0.62, specificity = 0.636), and TC (AUC: 0.620, sensitivity = 0.60, specificity = 0.673). Of note, there was a strong trend toward improved diagnostic accuracy for PD patients when these blood biomarkers were combined with Homocysteine and Urea (AUC: 0.779, Figures 3M, N), indicating that these selected blood biomarkers may be promising factors to differentiate PD from HC.

### Associations between blood biomarkers measure and progression to PD

**Figure 4:**
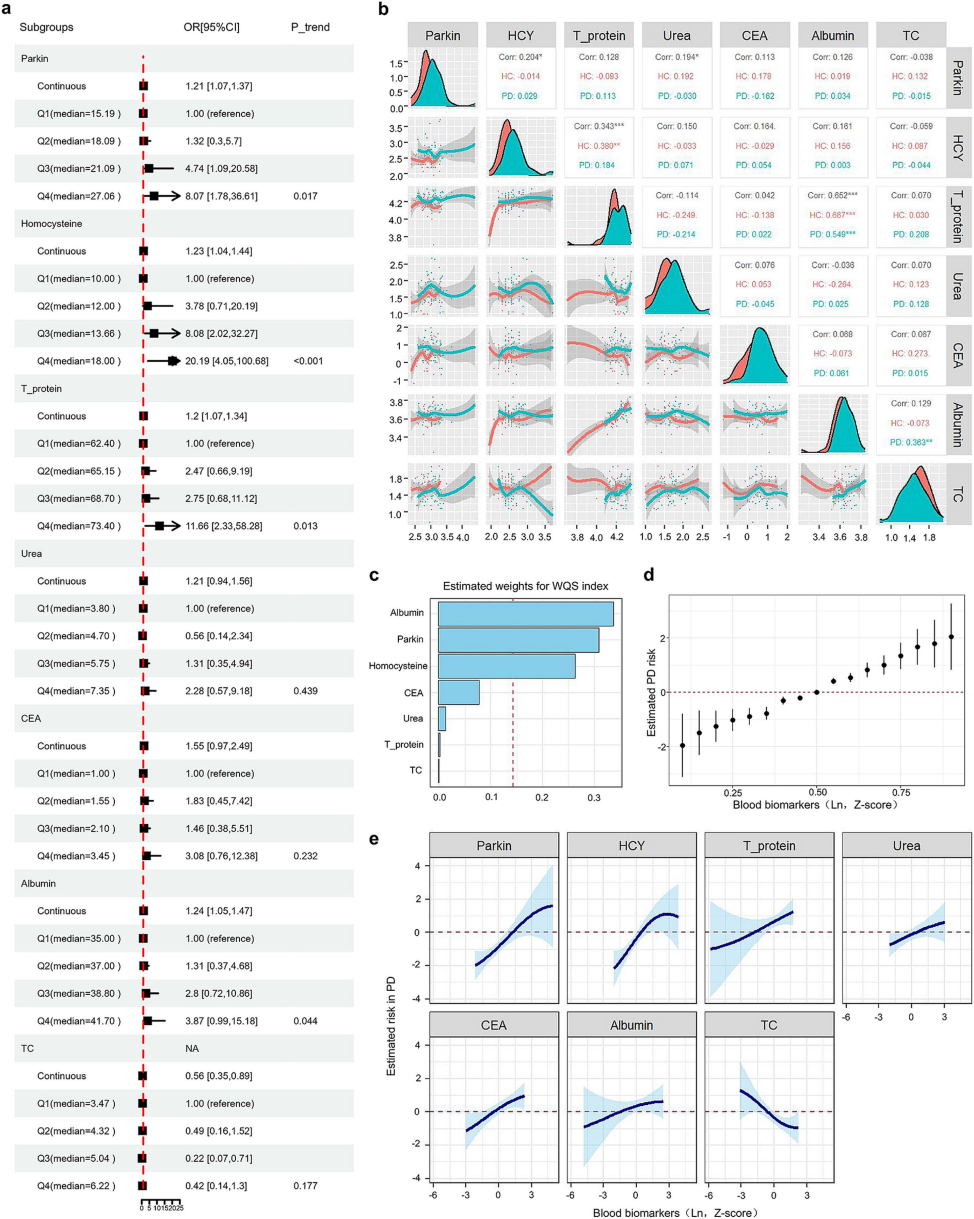
Associations between blood biomarkers and progression to PD. **(A)** Forest plots show binary logistic regression results for seven biomarkers, adjusted for age, sex, education, BMI, smoking, alcohol consumption, and histories of HP and DM. Subjects were categorized into four groups (Q1, Q2, Q3, Q4) based on biomarker quartiles, with individual levels replaced by group medians. **(B)** A multivariate correlation scatter matrix with LOESS analysis was used to assess relationships among blood biomarkers in PD and HC participants. **(C)** Weighted values for PD biomarkers were calculated using WQS models. **(D)** Associations between blood biomarkers and PD risk were estimated using BKMR. This figure illustrates the combined effects of blood biomarkers on PD risk. The plot shows the difference in PD risk and the 95% confidence interval (CI) when blood biomarkers are set at specific percentiles versus their medians. **(E)** Exposure-response functions illustrate the relationship between each blood biomarker and PD risk, with other biomarkers held at median values.

The analysis of the Binary logistic regression after adjusting for age, sex, education, BMI, hypertension, and diabetes mellitus revealed an increased odds ratio (OR) associated with interquartile range (IQR) increases in Parkin levels among PD participants (Q4/Q1 = 8.07, *p* for trend = 0.017, Figure 4A and Supplementary Table 5). Each quartile augment in IQR was associated with an obvious increase in the odds of incident PD (Q2/Q1 = 1.32, Q3/Q1 = 4.74, Figure 4A). Moreover, the IQR increment in Hcy, total proteins, and albumin levels were also significantly associated with the risk of subsequent PD diagnosis: the ORs were 20.19 (95% CI: 4.05–100.68, *p* for trend = 0.017) for Hcy, 11.66 (95% CI: 2.33–58.28, *p* for trend = 0.013) for total proteins, 3.87 (95% CI: 0.99–15.18, *p* for trend = 0.044) for albumin. By contrast, we did not find any statistical significance in the trend of PD risk for Urea (Q4/Q1 = 2.28, *p* = 0.439), CEA (Q4/Q1 = 3.08, *p* for trend = 0.232), and TC (Q4/Q1 = 0.42, *p* for trend = 0.177). Using locally weighted regression (LOESS) to examine the relationship among these selected blood biomarkers in PD and HC participants (Figure 4B), we found that Hcy showed a strong relationship with total proteins in HC subjects (*ρ* = 0.380; *p* < 0.001). We then analyzed the correlation of total proteins and other blood biomarkers in both groups and found that total proteins were positively associated with albumin (*ρ =* 0.652, *p* < 0.0001). No significant correlation was observed among Urea, CEA, TC and other biomarkers.

Next, to evaluate the association of selected blood biomarkers with PD risk evaluation. Notably, the Weighted Quantile Sum (WQS), a statistical model for multivariate regression in the high-dimensional dataset that operates in a supervised framework, was used to calculate a single score to evaluate the individual effect of the blood biomarkers on PD risk (Eggers et al., 2022). In this study, results from WQS analyses suggested that increased levels of albumin, Hcy, Parkin, and CEA were the highest four factors resulting in PD (Figure 4C and Supplementary Table 6). The same pattern of findings was observed by Bayesian kernel machine regression (BKMR). As represented in Figures 4D,E, the plots were applied to delineate the individual exposure-response functions for each blood biomarker and joint effects of blood biomarkers mixture on PD risk, after adjusting for age, sex, education, BMI, hypertension, and diabetes mellitus. Both BKMR and WQS models clearly demonstrated a positive dose–response trajectory in the association of Hcy, total proteins, Urea, CEA, and albumin and an increased risk of subsequent PD.

### Relationship with Parkin and blood biomarkers profile

To determine whether the levels of these blood biomarkers could be impacted by Parkin status, we constructed the groups divided into Parkin positive (+) and negative (−) according to the cutoff value. However, most blood biomarkers, such as albumin, Hcy, total proteins, and TC were similar between Parkin (+) and (−) groups (Supplementary Figures 2A–F), indicating that these blood markers were not associated with Parkin levels. Next, we compared the individuals with Parkin (+) to those with Parkin (−) at baseline and found higher Urea levels in the PD Parkin (+), and lower CEA concentrations in the PD Parkin (−) groups. We next sought to assess whether combining Parkin with these selected blood biomarkers could further improve the accuracy of PD diagnosis. Parkin and these blood biomarkers were included and removed step by step to assess their contribution to the model. The best model included blood Parkin, Hcy, total proteins, and Urea, with an accuracy of 0.841 (Supplementary Figure 2H). Moreover, it is notable that the model only incorporated measures of blood Parkin, total proteins, and Urea displayed a similar accuracy to that of the best model (AUC: 0.829). Of interest, when the model was constructed again to include two biomarkers, such as Parkin plus Hcy also provided a relatively high AUC of 0.817 (Supplementary Figure 2G). In summary, blood Parkin, in combination with Hcy, total proteins, and Urea, might significantly improve the diagnostic value of PD.

The above results indicated that Parkin was a significant risk factor for PD and associated with blood biomarkers including Hcy, CEA, Urea, total proteins, TC, and Albumin, especially Hcy, total proteins, and Urea. Therefore, we further explored whether these blood biomarkers could mediate the influences of Parkin on PD diagnosis (Supplementary Figures 2I–N). Mediation analyses with 10,000 bootstrapped iterations were carried out to examine the mediation effects of Parkin on PD. The results demonstrated that the relationship between Parkin and PD was partially mediated by CEA and albumin with the approximate proportion of mediation of 22.68% (*p* = 0.04) and 25.83% (*p* = 0.02), respectively, rather than Hcy (proportion: 7.62%, *p* = 0.36), total proteins (proportion: 6.55%, *p* = 0.06), Urea (proportion: 12.58%, *p* = 0.10), and TC (proportion: −23.47%, *p* = 0.18).

## Discussion

This cross-sectional study analyzed Parkin and various blood biomarkers in a large sample of idiopathic Parkinson’s disease (PD) patients and matched healthy controls. Key findings include: (1) PD patients had higher levels of blood Parkin, Hcy, total proteins, urea, albumin, and CEA compared to controls. Additionally, a model incorporating blood Parkin, Hcy, total proteins, and urea effectively distinguished PD from healthy controls, achieving a higher accuracy (AUC 0.841) than other biomarker combinations. (2) Gene set enrichment analysis suggested that pathways such as PINK1-Parkin-mediated mitophagy, urea cycle, cysteine degradation, and riboflavin metabolism may be involved in the Parkin mutation process. (3) Hazard models showed a positive dose–response relationship between Parkin, Hcy, CEA, and urea levels and the risk of developing PD, although Parkin levels did not significantly correlate with motor characteristics. The link between Parkin and PD was partially mediated by CEA and albumin, but not by Hcy, total proteins, or urea, which were unaffected by Parkin status. These results highlight the potential of blood biomarkers in the WBPLC cohort and suggest an effective PD diagnostic model using blood Parkin, Hcy, total proteins, and urea.

This study is the first to evaluate blood Parkin as a clinical biomarker for Parkinson’s disease (PD). We found that higher blood Parkin levels were linked to an increased risk of developing PD (Figure 4A). This finding was robust in a various analysis ways and models (WQS and BKMR), indicating minimal influence from confounding factors or reverse causation (Figures 4C–E). Additionally, only one prior study in a Japanese cohort indicated that blood Parkin levels could differentiate multiple sclerosis from neuromyelitis optica spectrum disorders (Cossu et al., 2021). PD has a long latency between biological onset and clinical symptoms, meaning some sporadic cases may be biologically active but not yet clinically evident at recruitment (Das and Ramteke, 2024). It’s unclear if blood Parkin is an early marker for preclinical PD or if the correlation is due to shared genetic factors. Previous studies have demonstrated that impaired mitophagy in PD triggers a cellular stress response, activating mitophagy-related genes, including Parkin (Liu et al., 2019). As the dysfunctional mitochondria accumulate in neurons, the demand for mitophagy increases, resulting in an upregulation of Parkin production (Joselin et al., 2012). Reactive oxygen species (ROS) may regulate Parkin expression, as ROS inhibitors can block Parkin recruitment in mouse embryonic fibroblasts and deleting the DJ-1 gene, which regulates ROS, increases stress-induced Parkin recruitment and mitophagy (Joselin et al., 2012).

Nuclear factor (erythroid-derived 2)-like 2 (NRF2) is a transcription factor that orchestrates the cellular response to oxidative stress, has been documented to enhance the expression of PINK1 under conditions of oxidative stress (Bonello et al., 2019; Chung et al., 2020), potentially facilitating the subsequent recruitment of Parkin and the upregulation of Parkin expression at the transcriptional level. Previous studies indicate that extracellular vesicles (EVs) contain all components of mitochondria (Zorova et al., 2022). Meanwhile, PRKN mutations are associated with an increased presence of extracellular mitochondria compared to control subjects, as evidenced by a clinical study (Choong et al., 2021). Consequently, the overproduction of Parkin and increased extracellular mitochondria leads to an excess release of Parkin into the bloodstream.

Notably, several preclinical studies have assessed changes in Parkin function in PD pathogenesis (Pickrell and Youle, 2015; Norris et al., 2015; Moskal et al., 2020). Established PD animal models are associated with abnormal Parkin-mediated mitophagy (Malpartida et al., 2021; Clark et al., 2021), driven by absolute impairment in mitochondria. Loss-of-function mutations in Parkin of *Drosophila* represent a grievous flight muscle defect resulting in locomotive behavioral problems and reduced lifespan (Pesah et al., 2004). Moreover, flies with Parkin mutations are more susceptible to oxidative stress and some dopamine neurons display abnormal shrinkage and morphology (Cha et al., 2005). However, the first Parkin-KO mouse models showed only mild phenotypes, such as the disruption of fine motor skills, slight abnormalities in dopamine metabolism and release, and no dopaminergic neuron loss (Goldberg et al., 2003). Another Parkin-null mouse model also did not cause motor behavioral phenotypes and DA neurodegeneration (Von Coelln et al., 2004). These results indicate that mice compensate for the loss of Parkin in DA neurons or that the neurons in mice do not reach a threshold of mitochondrial dysfunction necessary to cause detrimental phenotypes (Goldberg et al., 2003; Von Coelln et al., 2004).

In this cohort, higher baseline levels of Hcy, total proteins, urea, CEA, and albumin were linked to an increased risk of incident PD (Figures 4A,C–E). However, pre-existing health conditions that could affect these biomarkers were not accounted for, leaving potential residual confounding. To mitigate bias, we employed multiple risk models in our observational study. Taken together, the blood Hcy, CEA, and albumin levels could be used as indicators for reflecting the higher risk of subsequent PD diagnosis, which was supported by previous studies (Wang et al., 2017; Akil et al., 2015; Zhou, 2024; Fan et al., 2020). In a previous cohort study from China (Fan et al., 2020), blood Hcy levels in PD patients were elevated compared to those of HC. High Hcy drives PD development and progression while aggregating the clinical symptoms of PD patients (Zhou, 2024). That finding suggested that Hcy might be involved in the process of PD occurrence. Regarding the CEA, consistent with our results (Figure 3H), one cross-sectional study (Akil et al., 2015) including 51 PD patients and 50 healthy controls reported that the CEA was significantly higher in PD relative to the control group (mean 2.40 ± 1.51 vs. 1.72 ± 0.87 (ng/mL), *p* = 0.015). In contrast, one study noted that the levels of serum albumin were significantly lower in PD patients than those in controls (Wang et al., 2017). Multivariable logistic regression indicated that serum albumin is an independent risk factor for PD, with an AUC of 0.883 (95% CI 0.835–0.931) (Wang et al., 2017). Further research is needed to clarify the role of albumin in PD. Nonetheless, these results support the direct association between PD and blood levels of Hcy, CEA, and albumin.

This study’s key advantages include enrolling well-defined “typical” PD patients of varying severity and healthy controls, collecting detailed clinical and biospecimen data, and measuring multiple blood biomarkers simultaneously. We explored the relationships between blood Parkin and both motor and nonmotor variables, such as RBDQ-HK and UPDRS factors, which have been less studied.

### Limitations of the study

However, our study had limitations, including its cross-sectional design and lack of prospective follow-up. Blood levels of Parkin and other biomarkers were measured only at enrollment. Future research should track these biomarkers over time to better understand their changes in Parkinson’s disease. Moreover, the patients included in this study did not have genetic assessments. PD has many distinct pathophysiological pathways, the inclusion details clinically diagnosed PD, which could be highly heterogeneous. Second, the diagnosis of PD was not confirmed by postmortem pathological tests and may be susceptible to misclassification. Hence, there is a lack of comparison to any gold standard such as neuropathology limiting the validity of the “biomarker” application presented. Moreover, we assessed memory function only with MMSE, a simple measurement of global cognitive function. Third, although the blood Parkin level is significantly increased in patients with PD compared to controls in the cross-sectional design of comparison, the Binary logistic regression analysis revealed no correlation between disease severity and neuropsychological assessment. The possible reason may come from the relatively incomprehensive scales and inaccuracy evaluation. A future cohort with a larger sample size of participants and comprehensive assessment is warranted to confirm our findings and validate the role of blood Parkin in predicting disease features.

## Conclusion

Our results suggested that the blood Parkin level could serve as a minimally invasive, easily accessible biomarker for PD diagnosis. The model included blood Parkin, Hcy, total proteins, and Urea efficiently discriminated PD from HC with significantly higher accuracy.

## Data Availability

The data that support the findings of this study are available from the corresponding author upon reasonable request. Transcriptomic RNAseq datasets analysed in this study are available on NCBI GEO (Accession number: GSE90514, GSE7621 and GSE205450).

## Footnotes

^1^ http://software.broadinstitute.org/gsea/index.jsp

## Glossary

WPBLC: Wenzhou Parkinson’s Biomarkers and Living Characteristics study;
PD: Parkinson’s disease
HC: Healthy Control
BMI: Body Mass Index
HP: Hyper blood pressure
DM: Diabetes mellitus
LEDD: Levodopa Equivalents
UPDRS: Unified Parkinson’s disease rating scale
MMSE: Mini-Mental State Examination
HAMD: Hamilton Depression Scale
HAMA: Hamilton Anxiety Scale
RBDQ-HK: REM sleep behavior Disorder questionnaire-Hong Kong
ADL: Activity of Daily Living Scale
CSF: cerebrospinal fluid
NFL: neurofilament light chain
SNpc: substantia nigra pars compacta
CEA: Carcinoembryonic antigen
T_protein: Serum total protein
TC: Serum total cholesterol
Hcy: Homocysteine
asy-no: *α*-syn oligomers
p-asyn: phosphorylated α-syn
SD: Standardized deviation
IQR: interquartile range
RF: random forest
LASSO: Least Absolute Shrinkage And Selection Operator
AUC: Area under roc curve
OR: Odds ratios
WQS: Weighted quantile sum
BKMR: Bayesian kernel machine Regression
DE: direct effect
IE: indirect effect
TE: total effect
GEO: Gene Expression Omnibus
DEGs: differentially expressed genes
FC: Fold change
GO: Gene Ontology
GSVA: Gene Set Variation Analysis
GSEA: Gene Set Enrichment Analysis
KEGG: Kyoto Encyclopedia of Genes and Genomes
NRF2: erythroid-derived 2)-like 2
EVs: extracellular vesicles
