# Corrigendum: Parkin characteristics and blood biomarkers of Parkinson's disease in WPBLC study

**DOI:** 10.3389/fnagi.2025.1621994

**Published:** 2025-05-19

**Authors:** Haijun He, Xi Xiong, Yi Zheng, Jialong Hou, Tao Jiang, Weiwei Quan, Jiani Huang, Jiaxue Xu, Keke Chen, Jingjing Qian, Jinlai Cai, Yao Lu, Mengjia Lian, Chenglong Xie, Ji Luo

**Affiliations:** ^1^Department of Neurology, The Affiliated Huzhou Hospital, Zhejiang University School of Medicine (Huzhou Central Hospital), Huzhou, China; ^2^Department of Neurology, The First Affiliated Hospital of Wenzhou Medical University, Wenzhou, China; ^3^Department of Neurology, The First People's Hospital of Wenling, Taizhou, China; ^4^Department of Neurology, Yuhuan City People's Hospital, Taizhou, China; ^5^Department of Physiology, School of Medicine, National and Kapodistrian University of Athens, Athens, Greece

**Keywords:** Parkin, blood biomarkers, mitophagy, Parkinson's diseaseas, bioinformatics

In the published article, there was an error in the order of the **affiliation** section. Instead of “^1^Department of Neurology, The First Affiliated Hospital of Wenzhou Medical University, Wenzhou, China, ^2^Department of Physiology, School of Medicine, National and Kapodistrian University of Athens, Athens, Greece, ^3^Department of Neurology, The Affiliated Huzhou Hospital, Zhejiang University School of Medicine (Huzhou Central Hospital), Huzhou, China, ^4^Department of Neurology, Yuhuan City People's Hospital, Taizhou, China, ^5^Department of Neurology, The First People's Hospital of Wenling, Taizhou, China”, the affiliations should instead have been written as “^1^Department of Neurology, The Affiliated Huzhou Hospital, Zhejiang University School of Medicine (Huzhou Central Hospital), Huzhou, China, ^2^Department of Neurology, The First Affiliated Hospital of Wenzhou Medical University, Wenzhou, China, ^3^Department of Neurology, The First People's Hospital of Wenling, Taizhou, China, ^4^ Department of Neurology, Yuhuan City People's Hospital, Taizhou, China, ^5^ Department of Physiology, School of Medicine, National and Kapodistrian University of Athens, Athens, Greece.”

The corrected author list and affiliation numbers appear below.

“Haijun He^2, 5†^, Xi Xiong^1†^, Yi Zheng^2^, Jialong Hou^2^, Tao Jiang^2^, Weiwei Quan^2^, Jiani Huang^2^, Jiaxue Xu^2^, Keke Chen^2^, Jingjing Qian^2^, Jinlai Cai^2^, Yao Lu^2, 4^, Mengjia Lian^2, 3^, Chenglong Xie^2*^ and Ji Luo^1*^.”

The authors apologize for this error and state that this does not change the scientific conclusions of the article in any way. The original article has been updated.

