# Supplementary material for: Parkin characteristics and blood biomarkers of Parkinson’s disease in WPBLC study
Source: Front Aging Neurosci. 2025 Feb 26;17:1511272. doi: 10.3389/fnagi.2025.1511272 (PMC11897490; doi:10.3389/fnagi.2025.1511272)
Supplement: Supplementary file 1 [file Data_Sheet_1.pdf]

Supple materials

Supple information 1: Details about the WPBLC cohort

Supple Figure 1

Supple Figure 2

Supple Table 1: Selected blood biomarkers included in Subset 1 and two α-synuclein proteins detected in 234 participants

Supple Table 2: Basic Characteristics of PD patients and healthy controls in 2 subsets

Supple Table 3: Selected top blood biomarkers by Lasso and Random Forest in Subset 1

Supple Table 4: ROC analysis results of selected blood biomarkers for PD vs HC in Subset 1

Supple Table 5: OR (95% CI) in PD associated with blood biomarker levels with further adjustment for confounding factors in Subset 1

Supple Table 6: Estimated weights for WQS index in Subset 1

Supple information 1: Details about the WPBLC cohort

The WPBLC (Wenzhou Parkinson's Biomarkers and Living Characteristics study) cohort included 197 PD patients and 107 age-matched healthy controls from the First Affiliated Hospital of Wenzhou Medical University (March 2018 - October 2022). The study was approved by the hospital's Ethics Board (KY2021-153), and all participants gave written informed consent. Recruited patients with PD at the movement disorders clinic meet the MDS diagnostic criteria. All patients, aged over 50 and exhibiting late-onset symptoms, were treated with L-dopa alone or with dopamine agonists. None showed familial links or significant symptoms of atypical Parkinsonian syndromes, such as severe orthostatic hypotension or cerebellar signs. All participants were recruited at our specialized center for Parkinson's disease assessment and biospecimen collection. Healthy controls, matched by age and sex to PD subjects, included outpatients and inpatients without neurological disorders, Parkinsonism, psychiatric issues, brain trauma, stroke, or cancer. The WPBLC study standardized procedures to reduce pre-analytical variability in sample processing.

Supple Figures

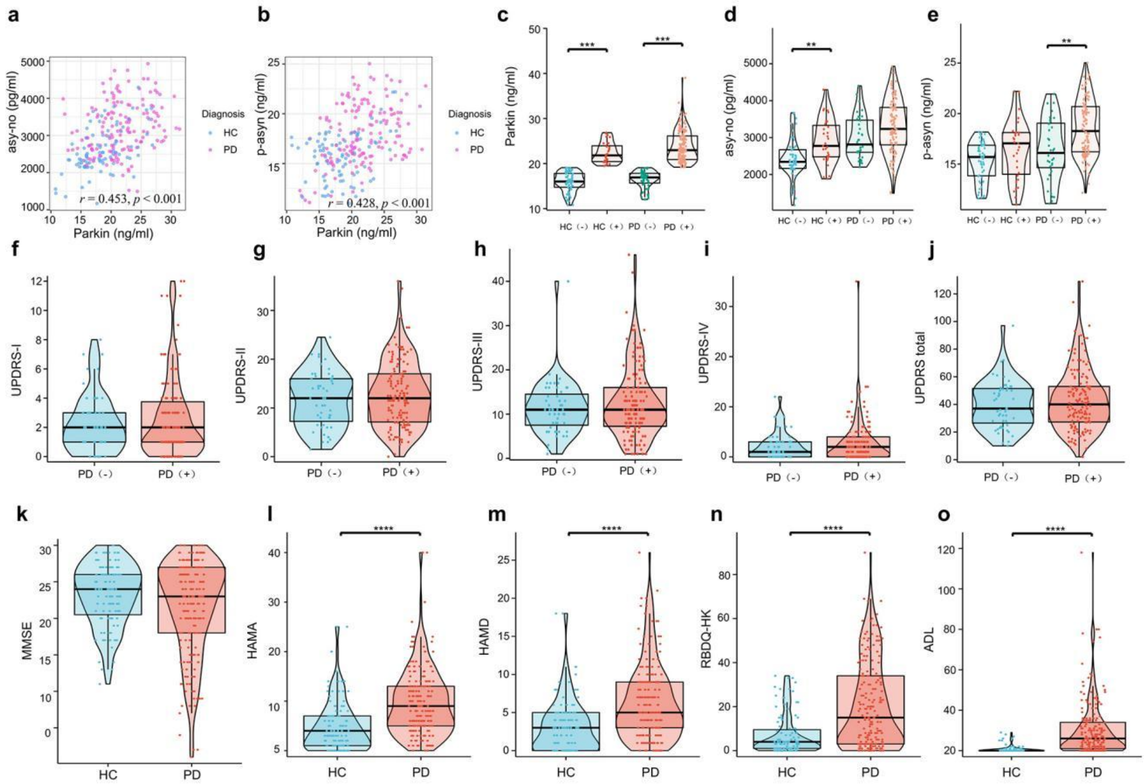

Supple Figure 1: (a-b) Nonlinear correlation analyses examined the relationship between blood levels of Parkin and a-synuclein proteins in 234 participants (148 PD and 86 HC). (c) The optimal cutoff for Parkin levels was established using the Youden index from ROC analysis, categorizing PD and HC subjects accordingly. (d-e) Levels of asy-no and p-asyn were compared across Parkin status subgroups in both PD and HC subjects. (f-o) Neuropsychological assessment results were compared between Parkin subgroups in PD and HC subjects. Statistical significance indicated as *$p < 0.05$, ** $p < 0.01$, *** $p < 0.001$, **** $p < 0.0001$ relative to the Parkin (-) group.

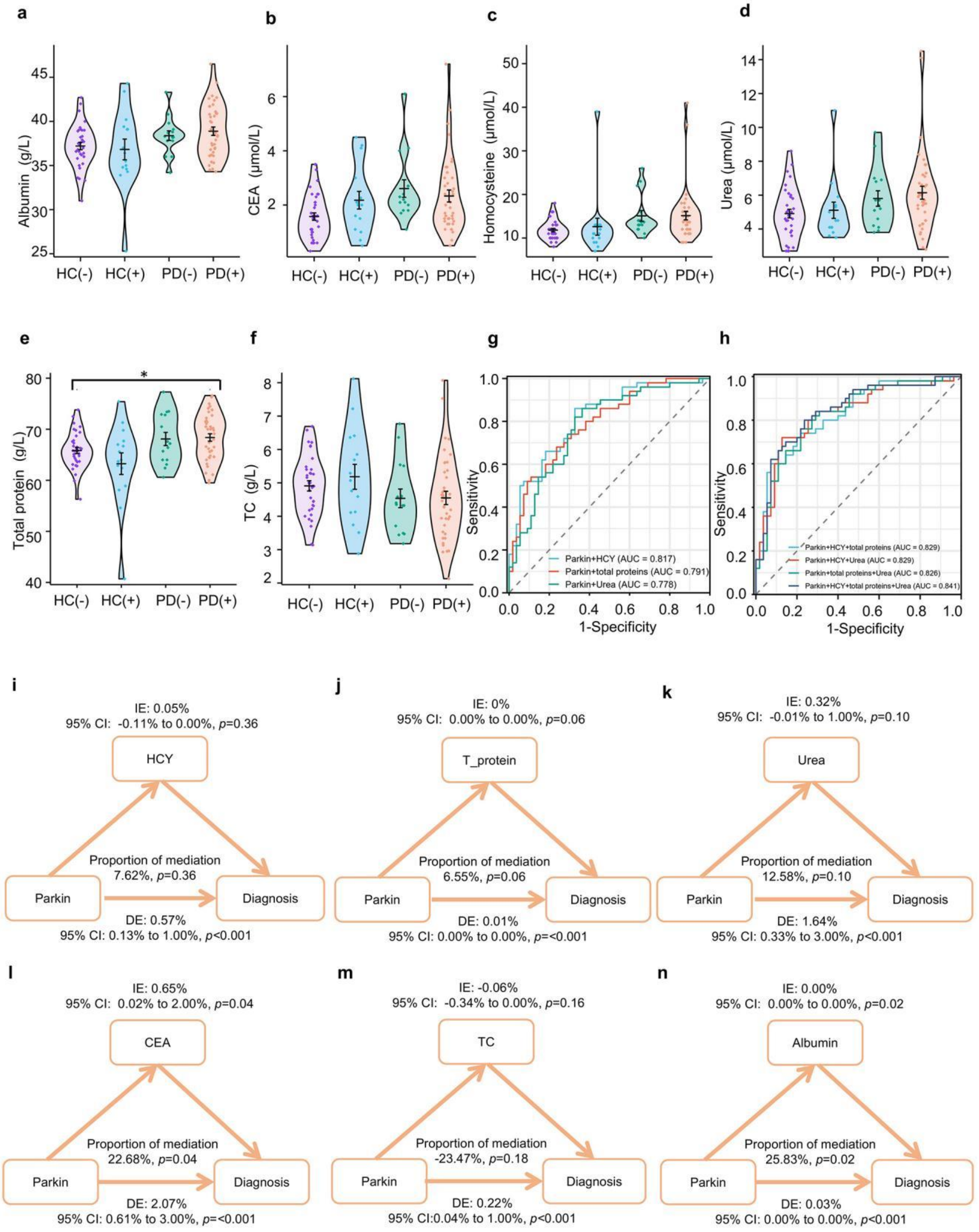

Supple Figure 2: (a-f) Showed six blood biomarker levels in PD and HC subjects based on Parkin status. (g, h) ROC analyses evaluated the predictive performance of models incorporating Parkin status and the top three blood biomarkers (HCY, total proteins, and Urea), with ROC curves and AUC values presented to assess their ability to differentiate PD status. (i-n) This plot shows the estimated proportion of the link between Parkin levels and PD risk mediated by other blood biomarkers.

Supple Table 1: Selected blood biomarkers included in Subset 1 and two α-synuclein proteins detected in 234 participants

Biomarkers	FC	log2(FC)	*p* value	AUC	AUC CI_lower	AUC CI_upper
Serum homocysteine level (umol/L)	1.256	0.328	0.003	0.749	0.656	0.842
Plasma Parkin (ng/ml)	1.288	0.365	0.000	0.742	0.648	0.836
Serum Total proteins (g/L)	1.050	0.070	0.001	0.668	0.564	0.771
Serum urea (mmol/L)	1.217	0.283	0.006	0.665	0.561	0.769
Serum carcinoembryonic antigen (CEA) (ng/ml)	1.372	0.456	0.006	0.654	0.550	0.759
Serum albumin level (g/L)	1.045	0.063	0.005	0.643	0.536	0.749
Serum total cholesterol level (mmol/L)	0.911	-0.135	0.049	0.620	0.512	0.728
Blood basophil percentage (%)	0.700	-0.515	0.076	0.610	0.503	0.718
Serum uric acid level (umol/L)	1.119	0.162	0.044	0.609	0.501	0.718
Serum Urea-Cr ratio	0.790	-0.341	0.249	0.607	0.496	0.717
Blood basophil count	0.015	-6.098	0.274	0.606	0.500	0.712
Prothrombin activity (%)	0.960	-0.058	0.061	0.603	0.495	0.712
Blood thrombocytocri	0.250	-2.002	0.061	0.600	0.490	0.711
Serum Creatinine kinase (CK) level (U/L)	0.933	-0.100	0.761	0.600	0.489	0.711
Serum total triglycerides level (mmol/L)	0.761	-0.394	0.030	0.598	0.489	0.708
Blood neutrophils percentage (%)	1.059	0.082	0.087	0.595	0.485	0.705
International normalized ratio (INR)	0.821	-0.284	0.355	0.591	0.482	0.701
Blood lymphatic percentage (%)	0.905	-0.144	0.092	0.591	0.481	0.700
Serum LDH level (U/L)	1.106	0.146	0.084	0.589	0.478	0.699
Blood neutrophils count	1.159	0.212	0.114	0.587	0.478	0.697
Serum total bilirubin level (umol/L)	1.149	0.201	0.034	0.585	0.476	0.695
Serum Cl (mmol/L)	0.994	-0.009	0.166	0.585	0.476	0.694
Blood mean corpuscular volume (fl)	1.037	0.052	0.056	0.585	0.474	0.696
Prothrombin time (PT) (s)	1.015	0.022	0.121	0.577	0.467	0.686
Blood mean platelet volume (fl)	0.957	-0.063	0.071	0.576	0.466	0.686
Serum globulin level (g/L)	1.050	0.071	0.051	0.572	0.462	0.683
APTT ratio	1.029	0.041	0.197	0.566	0.455	0.678
Activated partial thromboplastin time Activated partial thromboplastin time (ATPP) (s)	1.029	0.041	0.204	0.564	0.452	0.676
Blood mean corpuscular-hemoglobin concentration (g/L)	0.994	-0.008	0.343	0.564	0.452	0.676
Serum direct bilirubin	1.114	0.155	0.180	0.561	0.454	0.669

level (umol/L)						
Serum indirect bilirubin level (umol/L)	0.973	-0.039	0.850	0.558	0.448	0.668
Serum carbohydrate antigen 125 (CA125) (U/ml)	1.139	0.188	0.138	0.552	0.440	0.664
Blood lymphocyte count	0.956	-0.065	0.542	0.549	0.435	0.662
Blood leucocyte count	1.058	0.081	0.335	0.544	0.432	0.655
Plasma fibrinogen FIB (g/L)	0.988	-0.017	0.781	0.543	0.431	0.654
Serum thyroxine (nmol/L)	0.987	-0.019	0.737	0.541	0.430	0.653
Serum Low-density lipoprotein cholesterol (LDL-C) level (mmol/L)	0.947	-0.078	0.463	0.540	0.428	0.652
Serum aspartate transaminase (U/L)	1.060	0.084	0.596	0.539	0.428	0.651
Serum glucose (mmol/L)	1.061	0.086	0.223	0.539	0.428	0.651
Serum Free T4 (pmol/L)	0.975	-0.037	0.366	0.539	0.427	0.650
Blood hematocrit value (L/L)	0.217	-2.208	0.300	0.536	0.424	0.648
Serum K (mmol/L)	1.016	0.023	0.513	0.536	0.422	0.649
Serum alpha-fetoprotein (ng/ml)	0.937	-0.094	0.491	0.535	0.423	0.647
Blood red blood cell count	0.840	-0.252	0.278	0.535	0.423	0.646
Blood glycosylated hemoglobin (%)	1.025	0.035	0.493	0.532	0.421	0.643
Blood mononuclear percentage (%)	0.969	-0.046	0.577	0.527	0.415	0.639
Blood eosinophils count	1.067	0.094	0.712	0.527	0.415	0.639
Blood mononuclear count	1.011	0.015	0.876	0.526	0.413	0.639
Blood hemoglobin level (g/L)	1.014	0.020	0.520	0.524	0.412	0.637
Blood eosinophils percentage (%)	0.846	-0.241	0.345	0.524	0.412	0.635
Serum creatinine (umol/L)	1.034	0.048	0.503	0.521	0.409	0.632
Serum High-density lipoprotein cholesterol (HDL-C) level (mmol/L)	0.976	-0.035	0.598	0.520	0.408	0.632
Thrombin time (TT) (s)	1.001	0.001	0.941	0.517	0.405	0.629
Estimated glomerularfiltrationrate	1.032	0.045	0.452	0.516	0.404	0.628
Serum carbohydrate antigen 199 (CA199) (U/ml)	1.066	0.092	0.686	0.516	0.403	0.628
Serum ALT/AST ratio	0.294	-1.766	0.286	0.515	0.403	0.626
Serum glutamic-pyruvic transaminase (U/L)	0.878	-0.187	0.445	0.515	0.403	0.627
Blood platelet count	1.035	0.050	0.537	0.514	0.402	0.626
Plasma D-dimer (mg/L)	0.134	-2.903	0.064	0.512	0.398	0.626
Serum CKMB level (U/L)	1.002	0.003	0.982	0.510	0.399	0.622
Serum albumin-globulin ratio	0.992	-0.011	0.764	0.510	0.400	0.620
Serum Free T3 (pmol/L)	0.994	-0.009	0.871	0.508	0.396	0.621

Thrombin time ratio	0.996	-0.006	0.718	0.503	0.391	0.615
Serum Na (mmol/L)	1.019	0.027	0.296	0.503	0.392	0.613
Blood mean corpuscular hemoglobin (pg)	1.015	0.022	0.295	0.502	0.389	0.615
Serum Thyrotropin (mIU/L)	0.998	-0.003	0.929	0.502	0.390	0.614
Serum Triiodothyronine (nmol/L)	0.996	-0.005	0.929	0.502	0.390	0.614
asy-no (pg/ml)	1.2405	0.31089	0.000	0.754	0.690	0.818
p-asyn (ng/ml)	1.1461	0.19673	0.000	0.711	0.645	0.777

67 blood biomarkers were detected in Subset 1. Two α -synuclein proteins were detected in 234 participants (148 PD and 86 HC). Fold change (FC) and p values were calculated between PD group and HC group. All biomarkers were ranked by the AUC value. P values were obtained from t-test. AUC: area under roc curve. CI: confidence interval.

Supple Table 2: Basic Characteristics of PD patients and healthy controls in 2 subsets

Characteristics	Subset 1			Subset 2		
	HC (N = 50)	PD (N = 55)	*p* value	HC (N = 57)	PD (N = 142)	*p* value
Age (years)	66.0 [61.0;69.8]	68.0 [63.0;71.0]	0.365	64.0 [57.0;69.0]	66.0 [60.0;72.0]	0.130
Sex:			0.188			0.346
Female	31 (62.0%)	26 (47.3%)		31 (54.4%)	65 (45.8%)	
Male	19 (38.0%)	29 (52.7%)		26 (45.6%)	77 (54.2%)	
Height (cm)	160 (5.99)	159 (9.13)	0.524	160 [156;168]	160 [155;168]	0.576
Weight (kg)	62.5 (8.44)	62.1 (10.3)	0.831	65.0 [58.0;70.0]	60.0 [54.1;67.9]	0.066
BMI (kg/m2)	24.2 (2.81)	24.4 (2.98)	0.822	24.2 [22.7;26.7]	23.6 [21.5;25.6]	0.094
BMI Group:			0.609			0.642
<24	23 (46.0%)	27 (49.1%)		28 (49.1%)	80 (56.3%)	
24-28	23 (46.0%)	21 (38.2%)		8 (14.0%)	16 (11.3%)	
>28	4 (8.00%)	7 (12.7%)		21 (36.8%)	46 (32.4%)	
Education (years)	3.50 [0.00;5.00]	3.00 [0.00;6.00]	0.638	5.00 [1.00;8.00]	5.00 [0.00;8.00]	0.556
Disease History (years)	-	5.00 [2.00;7.00]	-	-	3.00 [1.00;6.75]	-
Smoker:			0.538			0.146
Current	6 (12.0%)	8 (14.5%)		7 (12.3%)	18 (12.7%)	
Former	2 (4.00%)	5 (9.09%)		0 (0.00%)	9 (6.34%)	
Never	42 (84.0%)	42 (76.4%)		50 (87.7%)	115 (81.0%)	
Drinker:			0.413			0.219
Current	5 (10.0%)	10 (18.2%)		8 (14.0%)	29 (20.4%)	
Former	4 (8.00%)	2 (3.64%)		0 (0.00%)	7 (4.93%)	
Never	41 (82.0%)	43 (78.2%)		49 (86.0%)	106 (74.6%)	
HP:			0.153			0.011
No	23 (46.0%)	34 (61.8%)		29 (50.9%)	101 (71.1%)	
Yes	27 (54.0%)	21 (38.2%)		28 (49.1%)	41 (28.9%)	
DM:			0.01			0.117
No	48 (96.0%)	42 (76.4%)		43 (75.4%)	122 (85.9%)	
Yes	2 (4.00%)	13 (23.6%)		14 (24.6%)	20 (14.1%)	
LEDD	-	375 [338;600]	-	-	388 [300;564]	-

67 blood biomarkers were detected in Subset 1 and plasma Parkin levels were detected in Subset 2. Continuous variables were assessed for normality by the Kolmogorov-Smirnov test, P-P plot and Q-Q plot. Data are expressed as mean (SD) (normal distribution), median [IQR] (abnormal distribution) or n (%) (categorical variable); *p* values of continuous variables obtained from t-test (normal distribution) or Mann-Whitney U test (abnormal distribution); *p* values of categorical variables obtained from chi-squared test. HC: Healthy Control; PD: Parkinson disease; BMI: Body Mass Index; Smoker (Current: people who have smoked continuously or cumulatively for 6 months or more and still smoke at the time of survey; Former: people who have smoked for more than 6 months and did not smoke at the time of survey; Never: people who have smoked for less than 6 months throughout their lives); Drinker(Current: people who have drinked alcohol continuously or cumulatively for 6 months or more and still drink at the time of survey; Former: people who have drinked alcohol for more than 6 months and did not drink at the time of survey; Never: people who have drinked alcohol for less than 6 months throughout their lives); HP: hyper blood pressure; DM: diabetes mellitus; LEDD: Levodopa Equivalents.

Supple Table 3: Selected top blood biomarkers by Lasso and Random Forest in Subset 1.

Biomarkers	HC (N = 50)	PD (N = 55)	*p* value
Homocysteine	11.0 [10.0;13.0]	13.7 [12.0;16.0]	<0.001
CEA	1.60 [1.02;2.22]	2.10 [1.50;2.90]	0.006
Urea	4.70 [4.00;5.88]	5.70 [4.50;6.85]	0.004
Total proteins	65.7 [62.9;67.7]	68.0 [64.6;72.0]	0.003
TC	4.95 [4.19;5.64]	4.40 [3.62;5.35]	0.034
Albumin	37.0 [35.3;39.0]	38.4 [36.6;40.8]	0.012

Data are expressed as median [IQR] and p-values of were obtained from Mann-Whitney U test. CEA: carcinoembryonic antigen; Total proteins: serum Total proteins; TC: serum total cholesterol.

Supple Table 4: ROC analysis results of selected blood biomarkers for PD vs HC in Subset 1

Biomarkers	AUC	CI	Cut-off value	Sensitivity	Specificity	Accuracy	Youden Index
Homocysteine	0.749	0.656 - 0.842	13.330	0.840	0.582	0.705	0.422
Total proteins	0.668	0.564 - 0.771	69.900	0.860	0.455	0.648	0.315
Urea	0.665	0.561 - 0.769	5.350	0.680	0.636	0.657	0.316
CEA	0.654	0.550 - 0.759	1.250	0.380	0.873	0.638	0.253
Albumin	0.643	0.536 - 0.749	37.750	0.620	0.636	0.629	0.256
TC	0.62	0.512 - 0.728	4.739	0.600	0.673	0.638	0.273

AUC: area under roc curve. CI: confidence interval.

Supple Table 5: OR (95% CI) in PD associated with blood biomarker levels with further adjustment for confounding factors in Subset 1

Biomarkers	Continuous OR (95% CI)	Q1 reference	Q2 OR (95% CI)	Q3 OR (95% CI)	Q4 OR (95% CI)	*p* for trend
Parkin	1.21 [1.07,1.37]	1.00 (reference)	1.32 [0.3,5.7]	4.74 [1.09,20.58]	8.07 [1.78,36.61]	0.017
Homocysteine	1.23 [1.04,1.44]	1.00 (reference)	3.78 [0.71,20.19]	8.08 [2.02,32.27]	20.19 [4.05,100.68]	<0.001
Total proteins	1.2 [1.07,1.34]	1.00 (reference)	2.47 [0.66,9.19]	2.75 [0.68,11.12]	11.66 [2.33,58.28]	0.013
Urea	1.21 [0.94,1.56]	1.00 (reference)	0.56 [0.14,2.34]	1.31 [0.35,4.94]	2.28 [0.57,9.18]	0.439
CEA	1.55 [0.97,2.49]	1.00 (reference)	1.83 [0.45,7.42]	1.46 [0.38,5.51]	3.08 [0.76,12.38]	0.232
Albumin	1.24 [1.05,1.47]	1.00 (reference)	1.31 [0.37,4.68]	2.8 [0.72,10.86]	3.87 [0.99,15.18]	0.044
TC	0.56 [0.35,0.89]	1.00 (reference)	0.49 [0.16,1.52]	0.22 [0.07,0.71]	0.42 [0.14,1.3]	0.177

The blood biomarkers were categorized into four quartiles (Q1, Q2, Q3, and Q4) as categorical variables. Multivariable logistic regression was applied to estimate odds ratios (OR) and corresponding 95% confidence interval (CI) for the associations of blood biomarkers PD risk. Models were adjusted for Age, Sex, BMI, Education, Smoker, Drinker, HP, DM. All *p* for trend were FDR-adjusted.

Supple Table 6: Estimated weights for WQS index in Subset 1

Blood biomarkers	Estimated weights for WQS index
Albumin	0.337
Parkin	0.309
Homocysteine	0.263
CEA	0.077
Urea	0.013
Total proteins	0.002
TC	0.000

Weighted quantile sum (WQS) regression was applied to explore the overall effects of blood biomarkers on PD as it performed well in characterizing environmental mixtures. The WQS index (ranged from 0 to 1) represented the mixed exposure level of blood biomarkers, and the components of concern were identified by non-negligible weights.
